# Molecular Mechanisms of Interaction of Human Serum Albumin with the CD36 Receptor: Insights from Molecular Dynamics Simulations

**DOI:** 10.3390/ijms27125395

**Published:** 2026-06-15

**Authors:** Daria A. Belinskaia, Richard O. Jenkins, Nikolay V. Goncharov

**Affiliations:** 1 Sechenov Institute of Evolutionary Physiology and Biochemistry, Russian Academy of Sciences, pr. Torez 44, St. Petersburg 194223, Russia; d_belinskaya@mail.ru; 2 Leicester School of Allied Health Sciences, De Montfort University, The Gateway, Leicester LE1 9BH, UK; 3 Research Institute of Hygiene, Occupational Pathology and Human Ecology of the Federal Medical Biological Agency, p.o. Kuz’molovsky bld.93, St. Petersburg 188663, Russia; 4 Department of Biological Chemistry, Petersburg State Pediatric Medical University, Ministry of Health of the Russian Federation, Litovskaya St. 2, St. Petersburg 194100, Russia

**Keywords:** albumin, receptor CD36, fatty acid transport, macromolecular docking, molecular dynamics

## Abstract

The rate of fatty acid (FA) uptake by cells depends on the presence of the CD36 receptor on the cell surface. However, unesterified FAs cannot circulate freely in plasma; they are bound to serum albumin. The molecular mechanisms of FA transfer from albumin to CD36 remain poorly understood. This study used macromolecular docking and molecular dynamics methods to investigate the interaction of the CD36 receptor with human serum albumin (HSA) loaded with oleic acid at the FA1-7 fatty acid-binding sites, with the aim of identifying potential mechanisms of FA transfer from HSA to CD36. The data obtained indicate that the interaction of HSA with CD36 does not result in direct FA transfer, but rather causes a local weakening of the affinity of individual FA sites on HSA. A comparative analysis was performed between the interaction interfaces predicted by macromolecular docking and those generated by AlphaFold 3. To further evaluate the influence of ligand nature, an additional molecular docking of HSA loaded with saturated (palmitic, PALM) and polyunsaturated (arachidonic, ARA) acids to the CD36 receptor was performed. This revealed a marked sensitivity of the protein–protein interface architecture to the type of lipid ligand, with the effect of ARA being more pronounced than PALM. Conversely, an alternative structure prediction using the AlphaFold3 algorithm demonstrated the opposite trend, indicating high geometric invariance and reproducibility of the complex. Ultimately, the proposed dynamic mechanism expands our understanding of the multi-stage processes governing FA transport across the endothelium.

## 1. Introduction

The CD36 receptor is known as scavenger receptor 3B (SR-B3), platelet membrane glycoprotein IV (GPIV), glycoprotein IIIb (GPIIIb), thrombospondin receptor, collagen receptor, fatty acid translocase (FAT), and even as an innate immune receptor [1]. Upon ligand binding, CD36 triggers a signaling cascade that mediates a wide range of proinflammatory responses [2,3]. CD36 is abundantly expressed in the microvascular endothelium, particularly in tissues with active lipid metabolism, such as the heart, skeletal muscle, and adipose tissue. In addition to the endothelium, CD36 is critically important and highly expressed in platelets, monocytes, smooth muscle cells, cardiomyocytes, and others, but is absent from the lymphatic vessels of the dermis [4]. An important function of endothelial CD36 is the transport of long-chain fatty acid (FA) molecules across the plasma membrane of endothelial cells lining the bloodstream. This process is particularly important in the capillaries of muscle tissue (skeletal and cardiac muscle) and adipose tissue, where the demand for FAs as an energy source is high. In addition to its normal physiological functions, CD36 plays an important role in fatty acid metabolism in cancer cells, where it serves as a key metabolic driver of tumor proliferation and metastasis [5].

CD36 acts in concert with fatty acid-binding proteins (FABPs) [6]. Based on their subcellular localization and functional role, fatty acid-binding proteins are divided into cytosolic (FABPc) and plasma membrane-associated (FABPpm). In humans, the FABPc family is represented by at least nine isoforms (FABP1–FABP9) [7]. It was shown that, in adipocyte–tumor cell interactions, CD36 directly interacts with FABP4, which mediates the import of FAs and their subsequent intracellular transport to various subcellular compartments [8]. FABPpm has been identified as mitochondrial aspartate aminotransferase (mAST), a classic enzyme of mitochondrial metabolism that is also found at the plasma membrane, where it acquires an additional function as a fatty acid-binding protein [9]. In contrast to FABP4, experimental data indicate that CD36 and FABPpm do not interact directly at the plasma membrane level and mediate fatty acid transport through independent mechanisms [10].

The rate of FA uptake depends on the presence of CD36 on the cell surface, which is regulated by subcellular vesicular recycling of CD36 from endosomes to the plasma membrane [6]. However, unesterified FAs cannot circulate freely in plasma; they are bound to human serum albumin (HSA) [11]. Quantitatively, serum albumin is the dominant protein in plasma, where its concentration is 500–700 μM. Along with other members of the albumin family, albumin acts as a carrier of endogenous and exogenous substances, including FAs [12]. The total concentration of major FA can reach 100 μM and even more; that is, their concentration is comparable to the concentration of HSA. FA binding has a significant effect on the conformation of albumin [13,14]. Beyond its physiological transport functions, the ability of albumin to bind hydrophobic molecules is widely utilized in clinical applications, such as the tumor-targeted delivery of nab-paclitaxel (Abraxane) [15], as well as newly developed lipid-modified prodrug complexes [16].

The molecular mechanisms of fatty acid transfer from albumin to CD36 remain poorly understood; it is unknown whether fatty acid transfer from albumin to CD36 involves their direct contact. Uncovering the structural details of albumin interactions at the cellular interface carries significant practical value, as it provides a molecular blueprint for optimizing lipid-based drug delivery platforms and designing targeted therapies to block CD36-mediated tumor progression. Albumin is known to act as a ligand for certain receptors and enzymes. In hyperglycemic environments, glycated albumin contributes to the pathogenesis of diabetes mellitus by binding to the receptor for advanced glycation end products (RAGE) on endothelial cells [17,18] and adipocytes [19]. Normally, albumin is an endogenous inhibitor of angiotensin-converting enzyme (ACE) [20]. When microvascular damage occurs, for example, in hyperglycemic environments, albumin can uncontrollably penetrate the blood–brain barrier and interact with TGFβ receptors [21]. The neonatal Fc receptor (FcRn), which is localized primarily within cells, is required for the delivery of newly synthesized albumin to the basolateral side of cells and subsequent albumin secretion into the bloodstream. The three-dimensional structure of the HSA complex with FcRn has been experimentally obtained [22]. Therefore, it is logical to assume that in the case of fatty acid transport, direct contact also occurs between HSA and CD36, after which the fatty acid molecule is translocated from the fatty acid-binding sites of albumin to the fatty acid-binding site on CD36. Confirmation of this assumption requires expensive biochemical and cellular approaches previously used to analyze direct and functionally significant interactions of CD36 with FABPs [8,9]. In the absence of experimental data, initial insights into the mechanism of albumin interaction with CD36 can be obtained using molecular modeling methods.

The aim of this study is to use macromolecular docking and molecular dynamics methods to investigate the interaction of HSA and CD36 and to identify probable mechanisms for fatty acid transfer from HSA to CD36.

## 2. Results

### 2.1. Building of the Three-Dimensional Models of HSA and C36

**Figure 1:**
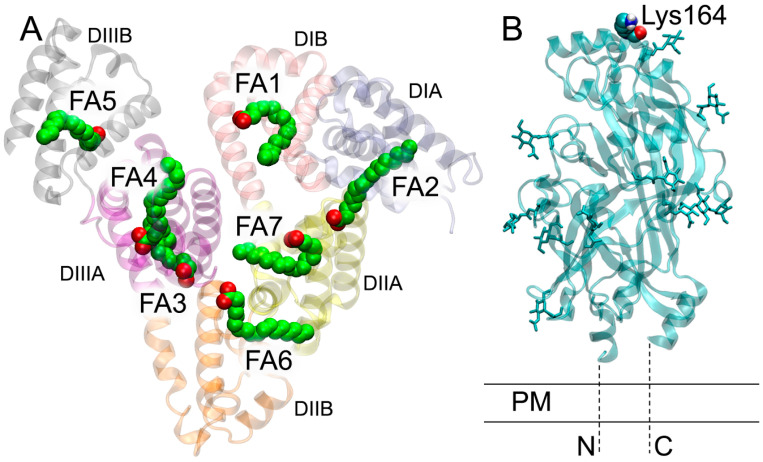
Structural organization of the HSA and CD36 molecules. (**A**) Structure of HSA in complex with seven oleic acid (OLA) molecules in the FA1-7 fatty acid-binding sites according to X-ray diffraction data (PDB entry 1GNI [13]). Missing fragments of OLA molecules were completed during model preparation using the CHARMM-GUI online service [24]). Domains DIA, DIB, DIIA, DIIB, DIIIA, and DIIIB are shown as gray, pink, yellow, orange, purple, and black stripes, respectively. OLA molecules are shown as spheres and highlighted in green. (**B**) CD36 structure based on X-ray crystallography (PDB entry 5LGD [23]). The glycosylated residues of CD36 are shown as sticks. Lys164, which serves as the entrance to the fatty acid tunnel, is shown as spheres. The solid black lines schematically represent the plasma membrane (PM). The dotted black lines schematically represent transmembrane fragments of CD36, which are absent in the 5LGD structure.

Experimentally obtained protein structures from the Protein Data Bank (PDB) were used as three-dimensional models of HSA and CD36. The HSA molecule is formed by a single polypeptide chain consisting of 585 amino acids. According to X-ray crystallography data, the tertiary structure of the protein is represented by three homologous domains, DI, DII, and DIII, and each of these domains consists of two subdomains, A and B, consisting of 6 and 4 helices, respectively (Figure 1A). Albumin is the main carrier of fatty acids in the circulatory system. The albumin molecule contains seven main fatty acid-binding sites, named FA1-7: FA1 in the DIB domain, FA2 at the border of the DIB and DIIA domains, FA3 in the DIIIA domain, FA4 in the DIIIA domain, FA5 in the DIIIB domain, FA6 in the DIIA and DIIB domains, and FA7 in the DIIA domain (Figure 1A). For modeling, the structure of the HSA complex with seven molecules of oleic acid (OLA, one of the four major fatty acids) at sites FA1-7 (PDB entry 1GNI [13], Figure 1A) was used.

CD36 is an integral membrane protein formed by a single polypeptide chain and comprising an extracellular domain, transmembrane helices, and short intracellular terminal regions. The 3D structure of the CD36 extracellular domain (Figure 1B), 400 amino acids in length, is available in the PDB and was used for modeling (PDB entry 5LGD [23]). It is known that lysine Lys164 and its surroundings (Ile157, Leu161, Lys166, Leu189, Pro191, and Tyr192) are the main entrance to the so-called “tunnel” for fatty acids inside the receptor (Figure 1B), and the region between the N- and C-terminal amino acids is the exit from the tunnel into the membrane and further into the cell (Figure 1B).

The 5LGD and 1GNI structures were prepared for further macromolecular docking using the CHARMM-GUI online service [24]. During the preparation process, unused ligands and water molecules were removed, and hydrogen atoms and missing fragments of the OLA molecules were added.

### 2.2. Interaction of HSA with CD36 According to Macromolecular Docking

**Figure 2:**
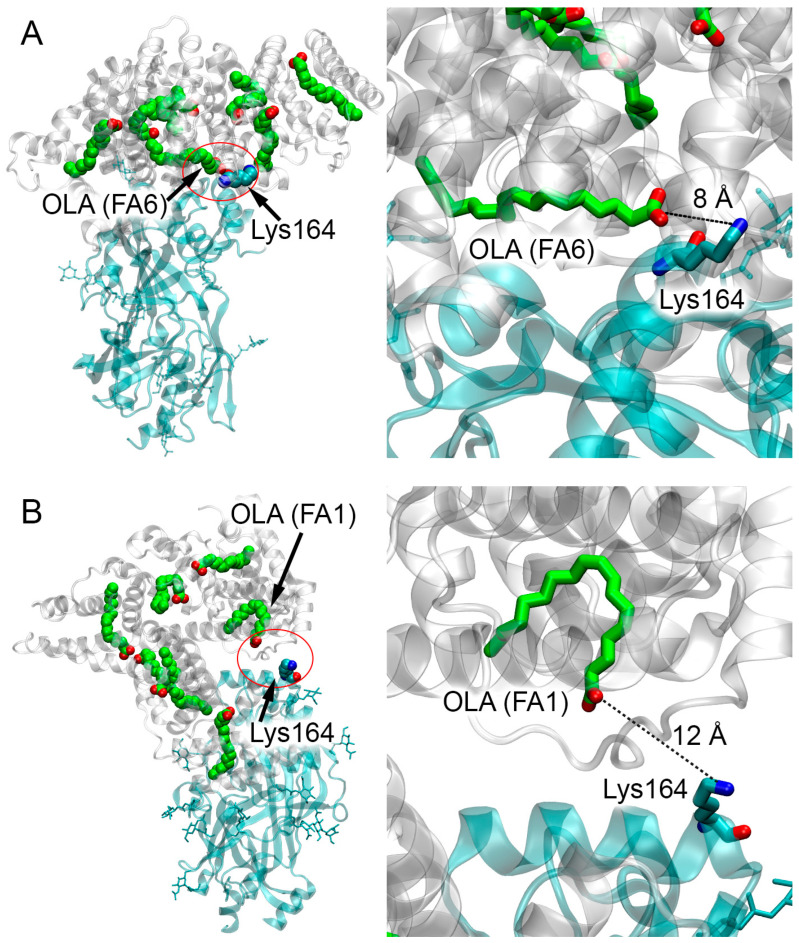
HSA-CD36 complexes with the minimum distance between the albumin-bound oleic acid (OLA) molecule and the entrance to the fatty acid tunnel in the CD36 receptor molecule (Lys164), according to macromolecular docking data performed using GRAMM (complex 1, (**A**)) and ZDOCK (complex 2, (**B**)). The HSA molecule is shown as a gray ribbon, and the CD36 molecule as a blue ribbon. The carbon atoms of the OLA molecules are highlighted in green. Glycosylated residues of CD36 are shown as sticks. The left panel displays the general view, and the right panel shows a detailed view. Red circles and arrows indicate the interaction regions between specific OLA molecules and the Lys164 residue. Dashed lines in the right panels represent the measured distances between the carboxyl groups of OLA and the amino group of Lys164.

Using the online programs GRAMM [25] and ZDOCK 3.0.2 [26], the HSA molecule loaded with seven OLA molecules was docked on the surface of the CD36 receptor. As the result of macromolecular docking procedures, 30 possible conformations of the HSA-CD36 complex were obtained (20 conformations in the GRAMM program and 10 conformations in the ZDOCK program). To test the hypothesis that the OLA molecule “slides” from the FA sites of albumin into the fatty acid tunnel of the CD36 molecule, conformations in which one of the OLA molecules in albumin was located as close as possible to the entrance to this tunnel on CD36, namely to Lys164, were selected for further analysis. Two such conformations are shown in Figure 2; complex 1 was obtained using the GRAMM program (Figure 2A), and complex 2 was obtained using the ZDOCK program (Figure 2B). In complex 1, the OLA molecule closest to Lys164 is bound at site FA6 (Figure 2A); and in complex 2, at site FA1 (Figure 2B). The distances between the carboxyl group of OLA and the cationic group of Lys164 are 8 and 12 Å in complexes 1 and 2, respectively. Table 1 presents the structural characteristics of complexes 1 and 2: the main amino acids of HSA and ACE involved in the contacts between the proteins (at a distance of no more than 3.5 Å), as well as the amino acids involved in specific interactions (hydrogen bonds and salt bridges).

According to the data obtained, in complex 1, domains DI and DII of albumin predominantly interact with the CD36 molecule, whereas in complex 2, it is domains DI and DIII.

### 2.3. Interaction of HSA with CD36 According to MD Simulations: Conformational Analysis

**Figure 3:**
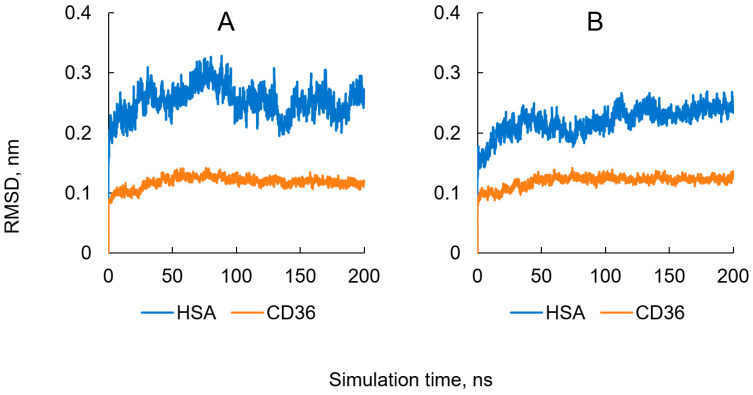
Time dependence of RMSD of Cα atoms of HSA (blue) and CD36 (orange) in complexes 1 (**A**) and 2 (**B**). The time evolution of RMSD values characterizes the structural stability of HSA and CD36, highlighting differences in their conformational behavior within the complexes. Each plot shows the averaged values obtained from three independent simulations for each complex.

Conformational changes in the complexes obtained by macromolecular docking were calculated using the method of molecular dynamics (MDs). Figure 3 shows the time dependence of the root mean square deviations (RMSDs) of Cα atoms of HSA and CD36 relative to the starting conformation. RMSD characterizes the stability of a protein molecule: if the RMSD value reaches a plateau during the simulation and does not increase further, this means that the system has reached a state of equilibrium and the structure is stable. In complex 1 (Figure 3A), the RMSD value of the Cα atoms of HSA increases in the first 70 ns of the simulation and then fluctuates at a level of 0.2–0.3 nm. In complex 2, the HSA molecule is less conformationally flexible: the RMSD value stabilizes after 100 ns of the simulation and fluctuates slightly around the mean value of 0.25 nm (Figure 3B). The CD36 molecule is less conformationally labile compared to HSA: in both complexes, the RMSD value of Cα atoms increases during the first 20 ns of the simulation and then stabilizes at 0.12 nm (Figure 3A,B).

**Figure 4:**
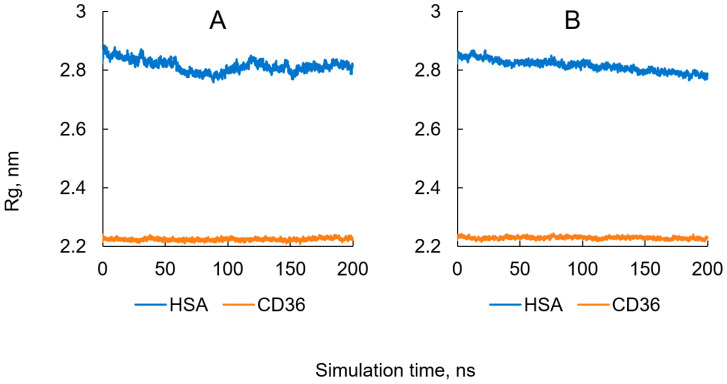
Time dependence of Rg of Cα atoms of HSA (blue) and CD36 (orange) in complexes 1 (**A**) and 2 (**B**). The variations in the Rg values reflect the fluctuations in protein compactness and the degree of its expansion or compression over time. Each plot shows the averaged values obtained from three independent simulations for each complex.

Figure 4 shows the time dependence of the radius of gyration (Rg) of HSA and CD36 molecules in complexes 1 and 2. Rg characterizes the degree of protein compactness; an increase in this parameter during the simulation process means an increase in the degree of protein unfolding, and vice versa. In complex 1, the Rg value of HSA decreases during 70 ns of the simulation and then fluctuates between 2.75 and 2.85 nm (Figure 4A). In complex 2, the Rg value of HSA decreases smoothly from 2.85 nm to 2.8 nm and stabilizes in the last 30 ns of the simulation (Figure 4B). Regarding the CD36 molecule, the Rg value of the receptor remains constant throughout the simulation in both complexes (Figure 4A,B).

**Figure 5:**
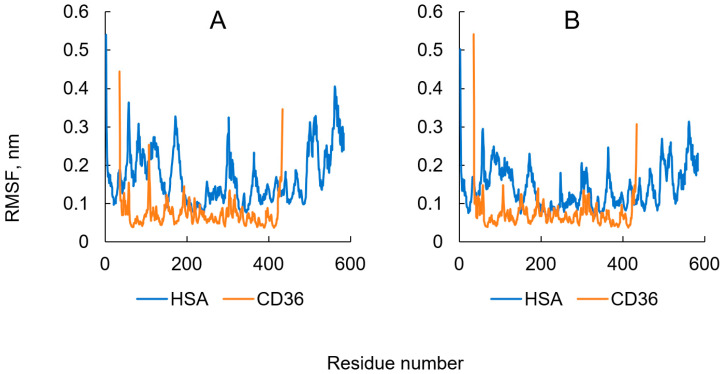
RMSF of Cα atoms of HSA (blue) and CD36 (orange) in complexes 1 (**A**) and 2 (**B**). The RMSF profiles illustrate the local residual mobility, highlighting flexible loops and rigid regions within the structures of HSA and CD36. Domain assignments for HSA residues are as follows: Domain I (residues 1–197), Domain II (residues 198–382), and Domain III (residues 383–585). In the CD36 structure, Lys164 and its neighboring residues define the entrance to the fatty acid translocation tunnel. Each plot shows the averaged values obtained from three independent simulations for each complex.

Figure 5 shows the values of the root mean square fluctuations (RMSFs) of Cα atoms of acids HSA and CD36 in complexes 1 and 2. The RMSF values characterize the local mobility of individual fragments of the protein molecule throughout the entire simulation. As can be seen from Figure 5, the HSA molecule in complex 1 has greater conformational lability than in the case of complex 2, which is consistent with the RMSD and Rg values (Figure 3 and Figure 4), with domains DI and DIII having the maximum mobility. As for CD36, in both complexes, the RMSF value of Cα atoms of the receptor is lower compared to that of HSA, which is also consistent with the RMSD and Rg values (Figure 3 and Figure 4) and indicates that the CD36 molecule is less conformationally labile compared to HSA.

The obtained data on RMSD, Rg, and RMSF values indicate that, during the simulation, the HSA molecule in complexes 1 and 2 exhibits different conformational behavior patterns. In complex 1, fast relaxation (50 ns) is replaced by stationary but high-amplitude fluctuations of both Rg and RMSD, thus characterizing this complex as conformationally labile. Complex 2 goes through a long compaction phase (up to 170 ns), after which the Rg value reaches a plateau with a minimal spread of values and a low amplitude of RMSD oscillations. This conformational behavior suggests that this complex represents an energetically favorable conformation characterized by a stable protein–ligand interface, whereas complex 1 appears to undergo further dynamic adjustment. The conformational behavior of the CD36 molecule is stable and does not depend on the albumin molecule.

### 2.4. Interaction of HSA with CD36 Receptor According to MD Simulation: Analysis of Binding Sites

**Figure 6:**
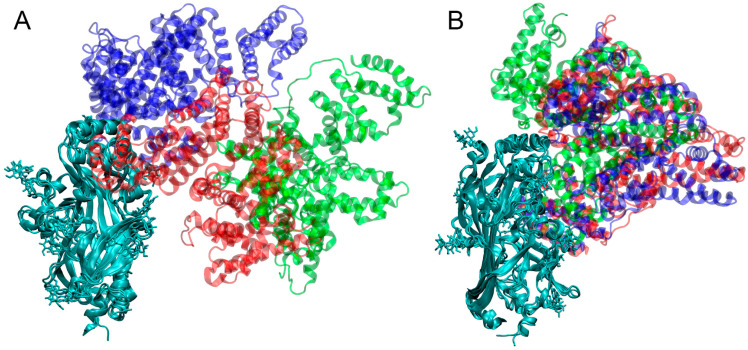
Final conformations of HSA-CD36 complexes obtained from three independent 200 ns MD simulations: (**A**) complex 1 and (**B**) complex 2. The CD36 molecule is shown in cyan for all simulations, and the albumin molecule is shown in three different colors (blue, red, and green), corresponding to each of the three simulations. The results demonstrate the structural stability and convergence of complex 2, in contrast to the divergent and unstable behavior observed for complex 1.

Conformational analysis of HSA-CD36 complexes described in the previous section showed that the CD36 molecule has low conformational lability compared to HSA, its structure is stable and independent of the albumin molecule. Therefore, for each of the studied complexes, we aligned the conformations of the complexes obtained from three independent 200 ns MD simulations using the CD36 structure as the basis for the alignment. The results are shown in Figure 6.

As can be seen from Figure 6, complex 1 is unstable: its conformational evolution is sensitive to the initial velocity distribution (which is unique for each of the three independent MD trajectories). Complex 2, in contrast, is stable, and its conformational behavior is reproduced in three independent simulations. Thus, the obtained result allows us to consider complex 2 as a more probable and representative model of the target HSA-CD36 complex.

**Figure 7:**
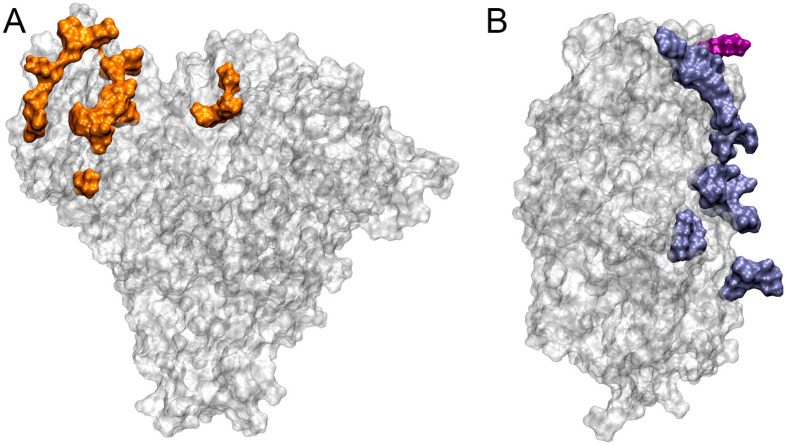
Sites of interaction between HSA and CD36 based on MD simulation: (**A**) sites on the surface of HSA and (**B**) sites on the surface of CD36 (Lys164 is highlighted in brighter color).

Table 2 and Figure 7 show the interaction sites between HSA (Figure 7A) and CD36 (Figure 7B) in complex 2 according to MD simulation. The list includes those amino acids that were involved in protein–protein contacts (distance between atoms ≤ 3.5 Å) in the final conformations in at least two of the three simulations performed. As can be seen from Table 2 and Figure 7A, domains DIB and DIIIB of HSA are involved in the interaction with CD36.

We then analyzed the distance between the carboxyl group of OLA bound in site FA1 and the cationic group of Lys164 in the three final conformations of complex 2, which was found to be 16 ± 3 Å (mean ± SEM, n = 3). Since, in the starting conformation, this distance was 12 Å (i.e., the distance did not decrease over 200 ns of simulation), this result indicates that, apparently, “direct transfer” of the OLA molecule from site FA1 of albumin to the CD36 receptor is not possible, and the transfer occurs via another mechanism. We hypothesized that HSA binding to CD36 weakens the affinity of FA for some FA sites, leading to a local increase in plasma FA concentrations and their subsequent interaction with CD36. Therefore, in the next step, we calculated the energy characteristics of the interaction of OLA with sites FA1-7 of both free albumin and albumin in complex with CD36.

### 2.5. Energetic Characteristics of the Interaction of HSA with CD36 According to MD Simulation

In this step, an additional block of three independent MD simulations was performed for the CD36-free HSA molecule loaded with seven OLA molecules. We then calculated the interaction energies (Es) of OLA molecules with sites FA1-7 of free and CD36-bound HSA (we studied only complex 2 since it is the most probable structure of the HSA-CD36 complex according to MD simulation). The value of E is the sum of the energies of Coulomb interactions and van der Waals forces calculated for short-range interactions. The results are presented in Table 3.

According to the data obtained, binding to CD36 has virtually no effect on interaction of OLA with FA sites of HSA. The only exception is a significant weakening of the OLA molecule’s affinity for site FA7 in complex 2. Thus, site FA7 is most susceptible to allosteric influence during albumin interaction with CD36.

### 2.6. Interaction of HSA with CD36 According to AlphaFold 3

Additionally, we used the AlphaFold 3 (AF3) service [27] to obtain possible conformations of HSA-CD36 complexes in the presence of seven oleic acid molecules (the parameters used are described in detail in Section 4.3). Five models of the HSA-CD36 complex were obtained (models AF3-1–AF3-5). For each model, we calculated the RMSD value between the computed structures of HSA and CD36, and the crystal structures of OLA-loaded HSA (structure 1GNI [13]) and CD36 (structure 5LGD [23]). The results are presented in Table 4.

**Figure 8:**
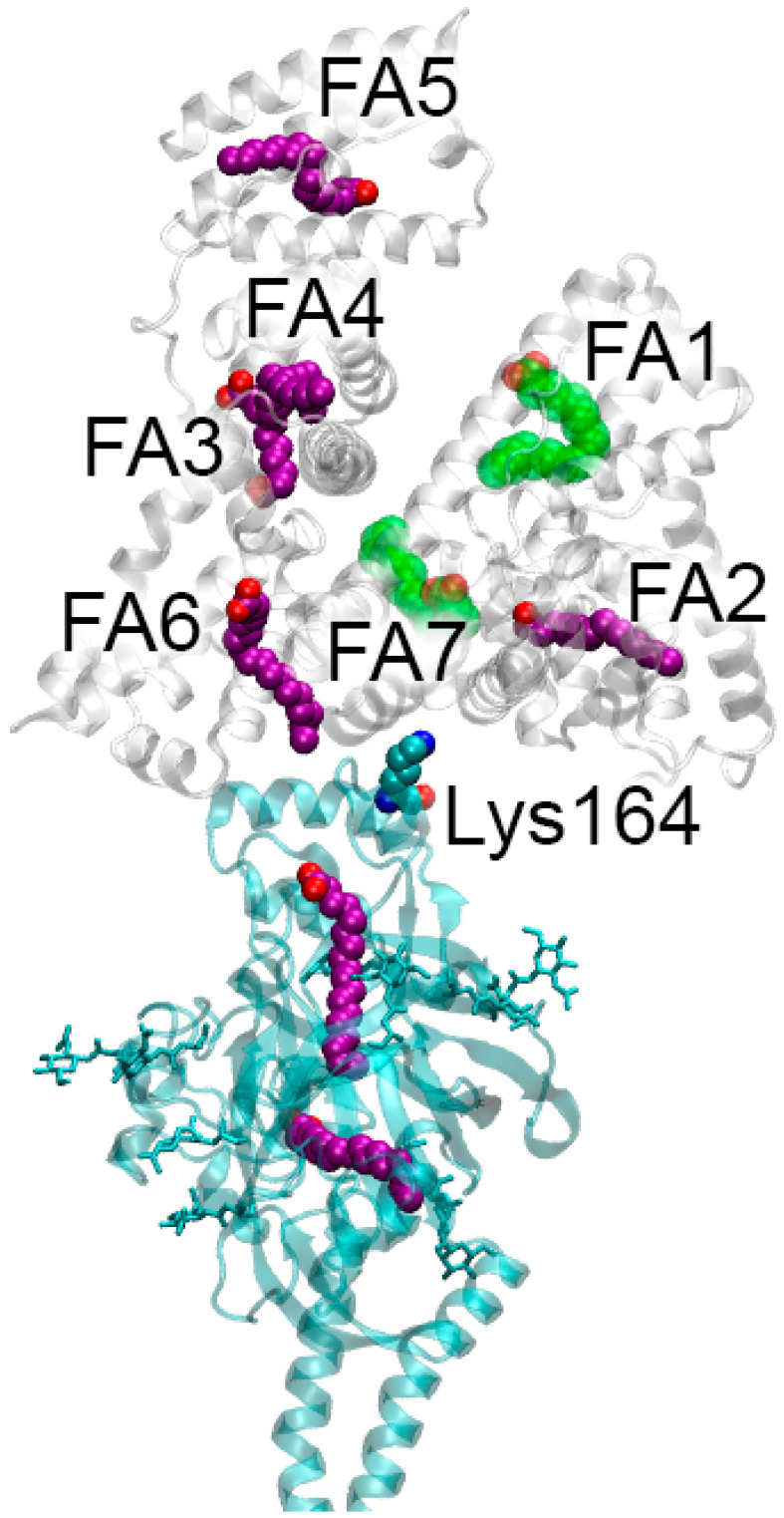
HSA-CD36 complex in the presence of 7 molecules of OLA obtained by AF3. The HSA molecule is shown as a gray ribbon, and the CD36 molecule as a blue ribbon. The OLA molecules in CD36 and in sites FA2-FA6 of HSA are highlighted in purple. The positions of the OLA molecules at sites FA1 and FA7 of HSA, according to X-ray diffraction data, are shown in transparent green. Lys164, and glycosylated residues of CD36 are shown as spheres and sticks, correspondingly.

As can be seen in Table 4, the optimal combination of RMSD values between the computed and crystal structures of HSA and CD36 was obtained for model AF3-1 (Figure 8). We selected this model for further analysis. The structure of model AF3-1 was optimized by the energy minimization method in the CHARMM27 force field [28] using the software GROMACS 2023.3 (University of Groningen, The Netherlands) [29]. We then analyzed the interaction sites between the proteins, and the results are presented in Table 5.

In model AF3-1, five OLA molecules are bound to HSA at sites FA2, FA3, FA4, FA5, and FA6, while sites FA1 and FA7 are free of OLA molecules. Two molecules of OLA in the resulting complex model are bound to CD36 instead of HSA (Figure 8). As for the interaction sites between the proteins, the area of their contact according to AF3 (Table 5) is approximately 3-fold less than according to macromolecular docking (Table 1). The amino acids of CD36 involved in the interaction with HSA according to AF3 (Table 5) are generally consistent with those according to macromolecular docking and subsequent MD simulations (Table 1 and Table 2). In the case of HSA, on the contrary, the results of docking and AF3 do not match. According to AF3 (Table 5), domains DIIA and DIIB of HSA are involved in the interaction with CD36, while according to docking and MD, domains DIB and DIIIB are involved (Table 1 and Table 2).

### 2.7. Interaction of CD36 with HSA Loaded with Arachidonic and Palmitic Acids According to Macromolecular Docking Data

The most stable complex of CD36 with OLA-loaded HSA (complex 2), described in Section 2.2, Section 2.3 and Section 2.4, was obtained using the ZDOCK 3.0.2 service and demonstrated conformational stability and reproducibility (Figure 6B). To analyze the influence of the fatty acid nature on the HSA-CD36 complex, we used the ZDOCK service to dock albumin loaded with palmitic acid (PALM, C16:0, a saturated fatty acid) and arachidonic acid (ARA, 20:4 (ω−6), a polyunsaturated acid) onto the surface of the CD36 receptor. The PDB structures 1GNJ [13] and 1E7H [30] were used as HSA-ARA and HSA-PALM models, respectively.

Among the obtained conformations of the HSA-ARA-CD36 and HSA-PALM-CD36 complexes, we selected conformations with the minimum RMSD value relative to complex 2. The RMSD value between complex 2 and the HSA-PALM-CD36 structure is 12 Å, and between complex 2 and the HSA-ARA-CD36 structure, it is 17 Å. Table 6 presents the sites of interaction between albumin and the receptor in the models studied.

**Figure 9:**
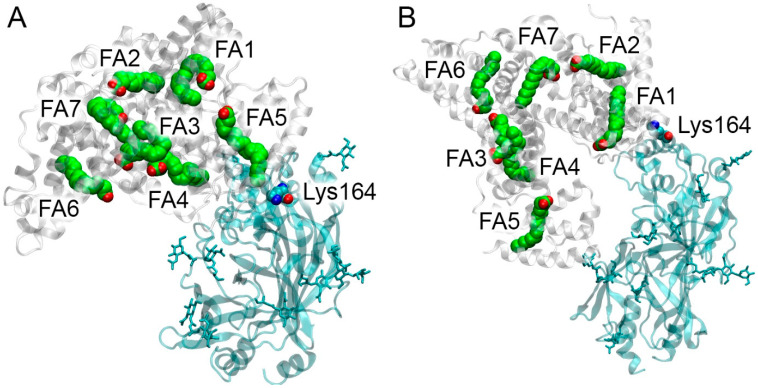
Complexes of CD36 with HSA loaded with ARA (**A**) and PALM (**B**) in FA sites according to macromolecular docking data performed using ZDOCK 3.0.2. The HSA molecule is shown as a gray ribbon, and the CD36 molecule as a blue ribbon. The carbon atoms of the ARA and PALM molecules are highlighted in green. Lys164 and glycosylated residues of CD36 are shown as spheres and sticks, correspondingly.

As can be seen from the RMSD values, visual inspection (Figure 2 and Figure 9), and interaction site analysis (Table 1 and Table 6), the nature of the fatty acid influences the architecture of the HSA-CD36 complex according to the macromolecular docking data.

The opposite result was obtained using AlphaFold3. The AlphaFold structure assembled from HSA, CD36, and seven PALM molecules was identical to the analogous structure for OLA-loaded HSA (Section 2.6, Figure 8), with an RMSD of 0.9 Å. As with the HSA-OLA-CD36 complex, the HSA-PALM-CD36 complex predicted by AlphaFold3 has five PALM molecules bound at sites FA2-FA6, sites FA1 and FA7 are free, and two PALM molecules are bound to CD36. Modeling of protein complexes with arachidonic acid is currently not supported by AlphaFold3.

## 3. Discussion

In the present work, we investigated the interaction of HSA and CD36 using molecular modeling methods in order to obtain preliminary data on possible mechanisms of fatty acid transfer from HSA to CD36. The primary hypothesis was that after HSA binds to the surface of CD36, a direct transfer of the fatty acid molecule from one protein to another (tunneling) occurs: the ligand slides from the binding site on albumin into a fatty acid tunnel inside the receptor, the entrance to which is the amino acid residue Lys164. This mechanism is found in nature. A classic example of tunneling is tryptophan synthase, an enzyme that catalyzes the final stages of tryptophan biosynthesis in bacteria, fungi, and plants. The enzyme structure includes α- and β-subunits that perform sequential reactions. In the active site of the α-subunit, indole-3-glycerol phosphate is cleaved to form indole, which is not released into the cytosol but instead moves directly through a hydrophobic tunnel approximately 25 Å long to the active site of the β-subunit. There, indole undergoes a PLP-dependent condensation reaction with L-serine, resulting in the formation of tryptophan [31]. Another example is the ceramide transfer protein (CERT). Its N-terminal START-domain physically contacts the membrane of the endoplasmic reticulum (ER), and the ceramide molecule passes from the cytosolic leaflet of the ER membrane into the protein’s hydrophobic pocket without entering solution, and then is transported to the Golgi apparatus [32]. However, our MD simulations of the OLA-loaded HSA complexed with CD36 showed that the ligand–Lys164 distance increases over time, even within the stable conformation. These findings suggest that, unlike the highly targeted mechanisms observed for tryptophan synthase or CERT, a direct and unassisted transfer of the fatty acid into the receptor tunnel is unlikely within the studied models.

Interestingly, according to macromolecular docking and MD simulations, complex 2 involves domains DI and DIII of albumin in interaction with CD36. A similar result was obtained in our computational experiments on the interaction of native HSA with angiotensin-converting enzyme [33], as well as earlier in the study of the structure of the HSA complex with FcRn using the X-ray diffraction method carried out by Oganesyan et al. [22]. According to MD and X-ray data, domains DI and DIII of albumin are also involved in the interaction with these proteins. The role of domains DI and DIII in the physiological activity of albumin has been noted by many researchers. For example, Paar et al. determined the structural features of albumin in patients with chronic liver disease [34]. Albumin in these patients is oxidized and overloaded with fatty acids and bilirubin. The authors demonstrated that albumin in healthy individuals has greater flexibility and mobility of domains DI and DIII. Fujiwara et al. used molecular dynamics to show that binding of myristate to HSA increases the mobility of domains DI and DIII, which in turn leads to an increase in the gyration radius of the protein molecule [35]. Ketrat et al. applied MD simulation to investigate the structural and dynamic properties of canine serum albumin and compared them with those of bovine and human albumins. It was found that the dynamics of domains DI and DIII determine the characteristics of each albumin [36]. Thus, the totality of the obtained data (stability and reproducibility of the structure of complex 2, and consistency with other computational and spectroscopic experiments, indicating the role of domains DI and DIII of albumin in the functional activity of the protein) allows us to consider complex 2 as a relevant model of the interaction of HSA with CD36.

When evaluating how the HSA-CD36 complexation affects fatty acid binding, we found that the calculated absolute interaction energies of OLA at FA sites do not correlate with experimental data. For example, it is known that sites FA2 and FA5 are the highest-affinity sites for binding long-chain FAs [37], whereas the calculated values of the interaction energies of OLA with these sites are not the most negative (Table 3). However, according to the data obtained, it was the FA2 and FA5 sites that were least affected by HSA binding to the receptor, which is consistent with experimental data on the strength of FA binding to these sites. According to the obtained calculated data (Table 3), a significant change in the interaction energy of OLA with albumin upon binding of HSA to CD36 occurs only at site FA7 in domain DIIA: in the HSA-CD36 complex, the oleic acid molecule interacts significantly weaker with site FA7 than in CD36-free albumin. This is consistent with the literature data. Site FA7 overlaps with the drug-binding site Sudlow I [38], which is known to be highly susceptible to allosteric modulation [39,40,41].

Domain DIIA, which contains site FA7, does not interact with the receptor surface in the HSA-CD36 complex we obtained. Therefore, it appears that when the OLA molecule’s affinity for this site weakens, it does not migrate directly into the receptor via the “common” hydrophobic corridor, but is released into the surrounding environment. That is, based on our data, we believe that the transfer of FA molecules from albumin to CD36 can be carried out by the following mechanism: the binding of HSA to CD36 leads to conformational changes in the albumin molecule and weakens the affinity of FA to some FA sites (presumably to site FA7). FA molecules are released from albumin and bind to CD36. It is not yet possible to determine how quickly this process occurs: whether there is a localized accumulation of FA molecules in the pericellular space, or whether the fatty acid molecule immediately binds to the receptor after release from albumin (and how to quantify this “immediately”). Theoretically, fatty acid accumulation can occur: the endothelial glycocalyx (EGL) provides the biophysical basis for this mechanism. It is a dynamic and compositionally heterogeneous “layer” between the membranes of endothelial cells, on the one hand, and blood components (plasma and cells), on the other hand. Structurally, EGL is a gel-like layer comprising oligosaccharide and polysaccharide chains of glycosaminoglycans, which are covalently linked to glycoproteins and proteoglycans [42]. Within EGL, local concentrations of solutes may differ from those in the central bloodstream. Therefore, an FA molecule released from albumin is not carried away by the bloodstream, but is retained in the dense pericellular space.

It is interesting to compare our proposed mechanism of FA transfer from HSA to CD36 in the pericellular space with the mechanism of FA molecule transfer from FABP1 to cytochrome P450 4A11 (CYP4A11, involved in FA metabolism) in the cytosol, described in the work [43]. According to the experimental data obtained by the authors, when FABP1 comes into contact with the enzyme, conformational changes occur in the carrier molecule, and its hydrophobic pocket partially opens. This reduces FABP1’s affinity for fatty acids, allowing the ligand to pass directly to the CYP4A11 active site without entering solution. However, the authors do not provide a quantitative estimate of the rate of this transition, just noting its contact-dependent nature.

The results obtained using AF3 are particularly noteworthy regarding the redistribution of OLA molecules within the predicted model AF3-1 (Figure 8). In the AF3-1 model, two OLA molecules migrated from sites FA1 and FA7 of albumin to bind directly with CD36. This suggests that the simulated HSA-FA-CD36 complex may represent an energetically favorable configuration for potential ligand transfer to the receptor. The evacuation of the FA1 and FA7 sites correlates with our MD data, which identified the FA7 site as one of the most sensitive to the presence of CD36. Although AF3 suggests an alternative interaction involving the DIIA and DIIB domains of HSA (contrasting with the DI and DIII domains identified by docking and MD), the entrance to the CD36 hydrophobic tunnel (Lys164) remains spatially dissociated from the primary FA sites on albumin in model AF3-1. These results are consistent with the hypothesis that FA transfer may proceed via local ligand release into the perireceptor space, rather than through a direct hydrophobic tunneling mechanism.

Our additional molecular docking of HSA loaded with saturated PALM and polyunsaturated ARA to the CD36 receptor revealed a marked sensitivity of the protein–protein interface architecture to the type of lipid ligand. The effect of ARA was more pronounced than that of PALM. Overall, this is consistent with the literature data indicating that the conformations of HSA when bound to mono- and polyunsaturated fatty acids are distinctively different [44]. However, an alternative structure prediction using the AlphaFold3 algorithm demonstrated the opposite trend, indicating high geometric invariance and reproducibility of the complex. Such a pronounced discrepancy between the results of classical docking and neural network modeling highlights the complexity of describing protein–protein interactions. Systematic analysis of dozens of docked conformations, testing their structural reproducibility and conformational behavior using multi-replica MD, represents a large-scale computational challenge that will be the subject of our upcoming research.

The discrepancy between the results obtained by docking and AlphaFold3 can be explained by the concept of encounter complexes [45,46]. According to this concept, during the first stage of their approach, proteins form a mobile ensemble of temporary configurations, which is maintained by long-range electrostatics and desolvation forces. The system then gradually transitions to a thermodynamically stable (“native”) state, which is fixed by numerous short-range contacts (hydrophobic interactions, hydrogen bonds, and salt bridges). Various computational approaches and algorithms can predict different states of this dynamic process: from early intermediates to the final and fully formed structure of the complex.

The primary limitations of this study stem from the inherent inaccuracies of macromolecular-docking scoring functions of ZDOCK and GRAMM programs used in the presented study, as well as the limitations of deep-learning-based confidence metrics in AlphaFold 3. Therefore, regarding the HSA-CD36 structures obtained in our work, we can state that these represent only possible conformations of the complexes. It is plausible that the predominant physiological conformation of the HSA-CD36 complex differs from those described herein. Since no computational tool possesses 100% predictive accuracy, future studies should reasonably include testing the stability of other identified conformations, as well as employing alternative docking engines with different scoring protocols to further expand the sampling landscape.

Among the limitations of the presented work, it can also be noted that we considered a rather simplified model of such a complex system as fatty acid transport. Our model included HSA, CD36, oleic acid, aqueous solution, and ions. In reality, FA transport across the endothelial barrier occurs in a more complex microenvironment. Within EGL, where possible contact of HSA with endothelial CD36 occurs, conditions of so-called molecular crowding are created—an environment with a high concentration of macromolecules in which the volume of available solvent is reduced, leading to a change in the physicochemical properties of biologically active substances. The influence of molecular crowding on the structure of many proteins [47,48,49], and on the thermodynamic and kinetic constants of many enzymes [50,51], has been experimentally shown. Therefore, it cannot be excluded that the affinity of fatty acids to albumin changes not only as a result of interaction with CD36, but also due to changes in the density of the surrounding medium. However, fully simulating such a multicomponent system currently represents a significant computational challenge. Even without taking the glycocalyx into account, a full-atom system comprising HSA, CD36, the lipid membrane, and solvent contains approximately 300,000–500,000 atoms. Inclusion of explicit glycocalyx models increases the system size to several million atoms, requiring the simultaneous deployment of thousands of processor cores [52,53]. For this reason, current working models are often limited to the isolated extracellular domain of CD36, since this is where key events in the uptake of fatty acid molecules from the extracellular space and their transport to the plasma membrane occur [54]. Nevertheless, with the rapid advancement of computing power and coarse-grained modeling algorithms [55,56], such large-scale calculations will likely become possible in the near future.

Moreover, we simulated the HSA-CD36 interaction at standard ionic strength and neutral pH. However, within the EGL, the abundance of negatively charged heparan sulfate and chondroitin sulfate chains can create a local proton gradient, so the pH in this environment can be lower than the systemic pH [57]. In the HSA-CD36 complex structure obtained by MD simulation, the protein–protein interaction is supported by three salt bridges (Glu501-Lys233, Asp512-Arg183, and Glu565-Arg183; Table 2). Changes in local ionic strength can cause electrostatic shielding of these charged pairs, while pH fluctuations are capable of changing the ionization state of functional groups, which together can critically affect the lifetime and stability of the formed complex [58]. It is known that the structure of albumin also depends on pH. At normal pH, the protein is in the so-called N-form (normal), but when the pH decreases below 5.0, it transforms into the F-form (fast), and when the pH increases above 8.0, it transforms into the B-form (basic). These conformational states differ primarily in their degree of structural compactness. It is known that when the pH decreases (N–F transition), the main conformational changes and loosening of the structure occur in domain III of albumin [59]. Since the major fatty acid-binding site FA5 is localized in domain III, it can be assumed that a change in pH will affect the affinity of the site for FAs. It has even been suggested that the N–F transition may play an important physiological role associated with the mechanisms of release and distribution of endogenous and exogenous ligands carried by albumin [60]. However, it has previously been shown that binding of fatty acids (in particular, PALM) stabilizes the N-form of albumin even in an acidic environment [61]. Therefore, it is possible that CD36, and not just the acidic environment, regulates the release of fatty acids from albumin in the juxtamembrane space. Answering this question requires further large-scale in vitro and in silico experiments simulating glycocalyx conditions.

In addition to CD36, albumin is also capable of interacting with another endothelial receptor—gp60 (a 60 kDa glycoprotein). It is a receptor with an unknown structure that regulates albumin transcytosis across the endothelium; its other name is albondin [62]. It is possible that HSA binding to gp60 may also influence the affinity of fatty acids for FA sites. The contribution of these effects is the subject of future research.

## 4. Materials and Methods

### 4.1. Macromolecular Docking

Macromolecular (protein–protein) docking involves the docking of one protein to the surface of another. Macromolecular docking of HSA to the surface of CD36 was performed using the online services GRAMM (Vakser Lab, the University of Kansas, KS, USA, https://gramm.compbio.ku.edu/, accessed 25 April 2026) [25] and ZDOCK 3.0.2 (ZLab, University of Massachusetts Medical School, MA, USA, https://zdock.wenglab.org/, accessed 25 April 2026) [26]. The GRAMM and ZDOCK services implement the ability to perform macromolecular docking of modified proteins (in the presented work, albumin loaded with fatty acids with glycosylated CD36). The scoring function in GRAMM is primarily based on geometric complementarity and smoothed empirical potentials [25]. The scoring function in ZDOCK is based on the interface atomic contact energy statistical potential, surface complementarity, and electrostatic interactions [26]. During the macromolecular docking procedure, the CD36 molecule was specified as the receptor, and the HSA molecule as the ligand. No other restrictions were applied. Visualization and structural analysis of the complexes were performed using the Visual Molecular Dynamics software package (VMD v1.9.4a53, University of Illinois Urbana-Champaign, IL, USA) [63].

### 4.2. Molecular Dynamics

Conformational changes in complexes HSA-CD36 were studied by MD simulation using software GROMACS 2023.3 (University of Groningen, Groningen, The Netherlands) [29] in CHARMM27 force field [28]. Each complex was virtually placed in a cubic periodic box filled with water molecules. The TIP3P water models (transferable inter-molecular potential with 3 points) was used to describe water molecules [64]. To neutralize the system, sodium ions were added. Temperature (300 K) and pressure (1 bar) were kept constant using the V-rescale thermostat [65] and Parrinello–Rahman barostat [66]. Long-range electrostatic interactions were treated by the particle-mesh Ewald method [67]. Lennard–Jones interactions were calculated with a cut-off of 1.0 nm. The LINCS algorithm (linear constraint solver for molecular simulations) was used to constrain bond lengths [68]. Before running the MD simulations, all the structures were minimized by steepest descent energy minimization and equilibrated under NVT (1000 ps) and NPT (5000 ps) ensembles. The time step for MD simulation was 0.002 ps. The length of the simulation was 200 ns. For each complex, three independent MD simulations were performed. Visualization and structural analysis of the final conformations of the complexes were performed using the Visual Molecular Dynamics software package (VMD v1.9.4a53, University of Illinois Urbana-Champaign, IL, USA) [63].

### 4.3. Constructing HSA-CD36 Complexes Using AlphaFold 3

The primary amino acid sequences of HSA and CD36 were inputted into the AlphaFold Server [27]. Glycan chains were added to the CD36 sequence according to the PDB structure 5LGD. Additionally, 7 molecules of OLA or PALM were added to the system as ligands. All other AF3 parameters were kept at default. Visualization and structural analysis of the complexes were performed using the Visual Molecular Dynamics software package (VMD v1.9.4a53, University of Illinois Urbana-Champaign, IL, USA) [63].

## Figures and Tables

**Table 1:** Interaction sites in HSA-CD36 complexes based on macromolecular docking data.

	HSA	CD36	HSA-CD36 Specific Contacts
Complex 1	Arg81, Glu86, Asp89, Lys93, Gln94, Glu97, Glu100, Cys101, Gln104, His105, Lys106, Asp108, Leu203, Gln204, Phe206, Gly207, Glu208, Arg209, Ala210, Lys212, Thr236, Thr239, Thr243, His247, Tyr319, Ala322, Lys323, Asp324, Leu327, Leu347, Lys351, Glu354, Glu358, Glu479, Ser480, Leu481, Val482, Asn483, OLA^FA6^	Pro73, Gln74, Met77, Met78, Asn79 *, Arg88, Gly89, Pro90, Tyr91, Pro124, Ser125, Ser127, Val128, Gly129, Thr130, Asp133, Asn134 *, Thr136, Met156, Asn163, Lys164, Ser165, Lys166, Ser167, Ser168, Phe170, Val172, Leu189, Glu397, Lys398, Gln400, Val401, Lys403, Asn404, Asn417 *	Gln94-Asn417 * (HB)
			Glu100-Arg88 (SB)
			Gln104-Tyr91 (HB)
			Gln104-Asp133 (HB)
			Glu208-Gln74 (HB)
			Arg209-Asn163 (HB)
			Arg209^HN^-Ser168 (HB)
			Lys212-Gln74 (HB)
			Thr236-Asn79 * (HB)
			Lys323-Glu397 (SB)
			Glu358-Lys398 (SB)
			Glu479-Lys164 (SB)
			OLA^FA6^-Lys166 (SB)
Complex 2	Asn109, Pro110, Asn111, Leu112, Pro113, Arg114, Val116, Arg197, Ser419, Pro421, Val424, Gln459, Val462, Leu463, Lys466, Thr467, Val498, Lys500, Glu501, Phe502, Asn503, Ala504, Glu505, Thr506, His510, Ile523, Lys524, Thr527, Lys573, Gln580	Asn102 *, Tyr149, Gln150, Asn151, Gln152, Phe153, Val154, Met156, Ile157, Asn159, Ser160, Leu161, Asn163, Lys164, Arg183, Pro191, Tyr192, Pro193, Thr195, Thr196, Tyr202, Pro203, Asn206, Thr207, Tyr230, Lys231, Ser237, Tyr238, Glu335, Glu397, Lys398, Gln400, Lys403	Asn109^O^-Lys398 (HB)
			Asn111-Glu397 (HB)
			Arg114^O^-Ser160 (HB)
			Glu501-Lys231 (SB)
			Glu501-Tyr238 (HB)
			Phe502^O^-Lys231 (HB)
			Asn503-Glu335 (HB)
			Glu505-Thr195 (HB)
			His510-Arg183 (HB)
			Lys524-Thr196 (HB)

*—glycosylated residues; HB—hydrogen bond; SB—salt bridge; OLA^FA6^—OLA molecule bound in site FA6 of HSA; the superscripts O and HN denote amino acids in which the backbone atoms participate in the interaction.

**Table 2:** HSA and CD36 interaction sites according to MD simulation.

HSA	CD36	HSA-CD36 Specific Contacts
Pro113, Arg114, Val116, Val498, Glu501, Phe502, Asn503, Glu505, His510, Asp512, Lys524, Glu565, Thr566, Ala569, Lys573, Ala577, Gln580	Asn102 *, Phe153, Val154, Ile157, Leu161, Lys164, Arg183, Tyr192, Pro193, Thr195, Thr196, Tyr202, Pro203, Asn205 *, Asn206, Lys231, Lys233	Glu501-Lys233 (SB)
		Phe502^O^-Lys231 (HB)
		Asp512-Arg183 (SB)
		Glu565-Arg183 (SB)
		Ala577-Asn102 * (HB)

*—glycosylated residues; HB—hydrogen bond; SB—salt bridge; the superscripts O denote amino acids in which the backbone atoms participate in the interaction.

**Table 3:** Energies (Es, kcal/mol) of interaction of OLA molecules with sites FA1-7 of free and CD36-bound HSA. The E value represents the sum of the energies of Coulomb interactions and van der Waals forces calculated for short-range interactions. Data are presented as the mean ± SEM from three independent simulations (n = 3).

Binding Site for FA	CD36-Free HSA	Complex HSA-CD36
FA1 (domain DIB)	−92.3 ± 3.8	−95.5 ± 1.4
FA2 (domains DIB and DIIA)	−97.7 ± 1.7	−96.4 ± 0.9
FA3 (domain DIIIA)	−106.4 ± 1.4	−103.7 ± 0.1
FA4 (domain DIIIA)	−89.3 ± 1.6	−93.7 ± 2.8
FA5 (domain DIIIB)	−78.0 ± 1.0	−79.8 ± 0.1
FA6 (domains DIIA and DIIB)	−76.7 ± 3.0	−74.0 ± 0.8
FA7 (domain DIIA)	−90.9 ± 1.1	−72.4 ± 3.1 *

*—differences from the control (free HSA) are significant, *p* < 0.05.

**Table 4:** RMSD values (Å) between computed and crystal structures of HSA and CD36.

AF3 Model of HSA-CD36 Complex	HSA (AF3/1GNI)	CD36 (AF3/5LGD)
Model AF3-1	0.503	0.389
Model AF3-2	0.609	0.380
Model AF3-3	0.615	0.419
Model AF3-4	0.504	0.399
Model AF3-5	0.606	0.385

**Table 5:** HSA and CD36 interaction sites according to AF3.

HSA	CD36	HSA-CD36 Specific Contacts
Glu227, Phe228, Ala229, Glu230, Ser232, Lys233, Thr236, Tyr263, Asn267, Asn318, Glu321, Val325, Phe326, Met329, OLA^FA6^	Asn151, Phe153, Ile157, Ser160, Leu161, Lys164, Leu189, Pro191, Tyr192	Glu227-Ser160 (HB)
		Glu230-Lys164 (SB)
		Asn267-Lys164 (HB)
		Glu321-Asn151 (HB)

HB—hydrogen bond; SB—salt bridge; OLA^FA6^—OLA molecule bound in site FA6 of HSA.

**Table 6:** Interaction sites of CD36 complexes with HSA loaded with ARA and PALM according to macromolecular docking data.

Model of HSA	HSA	CD36	HSA-CD36 Specific Contacts
HSA-ARA	Gly85, Glu86, Gln417, Ser419, Pro421, Thr422, Glu425, Leu463, Thr467, Pro468, Val469, Lys500, Glu501, Phe502, Asn503, Lys534	Tyr149, Asn151, Phe153, Val154, Met156, Ile157, Lys164, Leu189, Pro191, Tyr192, Asn321 *	Glu86-Asn321 * (HB)
			Glu86 ^HN^-Asn321 * (HB)
			Ser419-Phe153 ^O^ (HB)
			Lys500-Leu189 ^O^ (HB)
HSA-PALM	Pro35, Phe36, Glu37, Asp38, Thr79, Thr83, Pro113, Arg114, Leu115, Val116, Pro118, Glu119, Val122, Ala126, Asn130, Thr133, Phe134, Lys137, Tyr138, Tyr140, Glu141, Arg145, His510	Asn102 *, Phe153, Met156, Ile157, Ser160, Leu161, Lys164, Lys166, Arg183, Pro185, Ser188, Leu189, Pro191, Tyr192, Pro193, Val194	Glu37-Ser160 (HB)
			Val116 ^HN^-Pro191 ^O^ (HB)
			Ala126 ^O^-Lys164 (HB)
			Asn130 ^O^-Lys164 (HB)
			Lys137-Ile157 ^O^ (HB)
			Lys137-Ser160 (HB)

*—glycosylated residues; HB—hydrogen bond; the superscripts O and HN denote amino acids in which the backbone atoms participate in the interaction.

## Data Availability

The data presented in this study are available from the corresponding authors upon reasonable request.

## Abbreviations

The following abbreviations are used in this manuscript:

ACE	Angiotensin-converting enzyme
AF3	AlphaFold 3
ARA	Arachidonic acid
CYP4A11	Cytochrome P450 4A11
E	Energy of interaction
EGL	Endothelial glycocalyx
ER	Endoplasmic reticulum
FA	Fatty acid
FA1-7	Fatty acid-binding sites in albumin
FABP	Fatty acid-binding protein
FABP1–FABP9	Nine isoforms of FABP
FABPc	Cytosolic FABP
FABPpm	Plasma membrane-associated FABP
FcRn	Neonatal Fc receptor
HB	Hydrogen bond
HSA	Human serum albumin
NPT	Constant number of particles, N; pressure, P; and temperature, T
NVT	Constant number of particles, N; volume, V; and temperature, T
OLA	Oleic acid
PALM	Palmitic acid
PDB	Protein data bank
PM	Plasma membrane
Rg	Radius of gyration
RMSD	Root mean square deviation
RMSF	Root mean square fluctuation
SB	Salt bridge
